# Heterostructured WO_3_–TiVO_4_ thin-film photocatalyst for efficient photoelectrochemical water splitting

**DOI:** 10.1016/j.heliyon.2024.e25446

**Published:** 2024-01-28

**Authors:** Manal Alruwaili, Anurag Roy, Mansour Alhabradi, Xiuru Yang, Hong Chang, Asif Ali Tahir

**Affiliations:** a Solar Energy Research Group, Environment and Sustainability Institute, Faculty of Environment, Science and Economy, University of Exeter, Penryn, TR10 9FE, United Kingdom; b Physics Department, Jouf University, P.O. Box 2014, Sakaka, 42421, Saudi Arabia; c Department of Physics, Majmaah University, Majmaah, 11952, Saudi Arabia; d Department of Engineering, Science and Economy, University of Exeter, Exeter, EX4 4QF, United Kingdom

**Keywords:** Heterostructure, Photoelectrochemical, Photoanode, WO_3_, TiVO_4_, Thin film

## Abstract

Photoelectrochemical water splitting via solar irradiation has garnered significant interest due to its potential in large-scale renewable hydrogen production. Heterostructure materials have emerged as an effective strategy, demonstrating enhanced performance in photoelectrochemical water-splitting applications compared to individual photocatalysts. In this study, to augment the performance of sprayed TiVO_4_ thin films, a hydrothermally prepared WO_3_ underlayer was integrated beneath the spray pyrolised TiVO_4_ film. The consequent heterostructure demonstrated notable enhancements in optical, structural, microstructural attributes, and photocurrent properties. This improvement is attributed to the strategic deposition of WO_3_ underlayer, forming a heterostructure composite electrode. This led to a marked increase in photocurrent density for the WO_3_/TiVO_4_ photoanode, reaching a peak of 740 μA/cm^2^ at an applied potential of 1.23 V vs RHE, about nine-fold that of standalone TiVO_4_. Electrochemical impedance spectroscopy revealed a reduced semicircle for the heterostructure, indicating improved charge transfer compared to bare TiVO_4_. The heterostructure photoelectrode exhibited enhanced charge carrier conductivity at the interface and sustained stability over 3 h. The distinct attributes of heterostructure photoelectrode present significant opportunities for devising highly efficient sunlight-driven water-splitting systems.

## Highlights

• n-n type heterostructured WO_3_–TiVO_4_ thin-film photocatalyst for water splitting.
• Analyzed through various optical, structural and photoelectrochemical measurements.
• At 1.23 V, the photocurrent density is 9 times higher than pristine TiVO_4_.

## Graphical abstract

**Figure:**
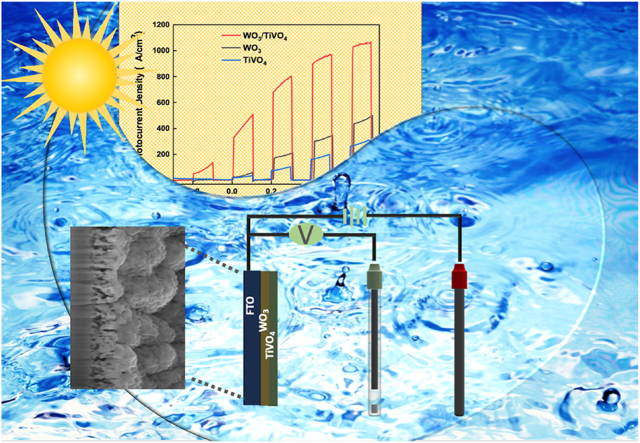

## 1 Introduction

The advancement of novel photocatalytic semiconductor materials and technologies has resulted in significant progress in elucidating the mechanisms governing energy device functionality [[1], [2], [3]]. Intensive scientific research has been dedicated to exploring the potential of large-scale energy storage or production applications to harness unlimited solar energy. Noteworthy advancements in this field include the exemplary performance and long-term stability demonstrated by supercapacitors, lithium batteries, and photoelectrochemical (PEC) water-split cells [4,5].

Although significant scientific advancements have been made in the past decade, photocatalytic hydrogen production has not been widely adopted, primarily due to its low quantum efficiency. PEC water splitting emerges as a paramount technique for hydrogen production, primarily because it harnesses inexhaustible solar energy, resulting in no carbon emissions or atmospheric pollutants. Solar energy is captured, converted, and stored in chemical bonds, yielding hydrogen, a potential storage fuel. Hydrogen is an environmentally friendly alternative fuel due to its high energy density of 143 MJ kg^−1^ [6]. During the combustion process, it is derived from water, which contributes to preserving human health and the environment. Researchers have been diligently working on advancing PEC applications since its inception in 1972, using TiO_2_ n-type photoanodes to facilitate comprehensive water-splitting reactions [7]. Confronted with a growing need for sustainable energy and recognizing the constraints of current materials in minimizing reliance on carbon-based sources, the scientific community has been actively engaged in the systematic identification and development of materials that can efficiently capture and utilize solar energy.

In various metal oxides, the valence band (VB) typically exhibits a significantly positive potential, posing challenges in the development of a semiconductor characterized by a narrow band gap. This is essential for achieving optimal optoelectronic absorption and ensuring the presence of a suitably negative conduction band (CB). Additionally, certain metal sulfides with narrow band gaps, such as CdS, present advantageous band edge positions for water splitting. However, the inherent instability of these photocatalysts renders them susceptible to photo-corrosion upon exposure to visible light in aggressive electrolytic environments [8]. To surmount these challenges, visible-light-driven photocatalysts must possess specific attributes, including appropriate band gaps, favourable band edge configurations, rapid charge kinetics, effective charge-carrier separation, and prolonged stability in aqueous surroundings. These features are imperative for realizing the most efficient water-splitting process.

According to the basic principle of achieving high performance of PEC water splitting, that has to fulfil some fundamental considerations such as required potential, charge carriers pathway, surface's state and recombination centres [9]. However, some existing materials showed low or wide band gaps that exhibited limited light harvesting, poor charge transport or are likely prone to photo-corrosion, fast charge carriers' recombination rate, and high trapping rate of charge carries at the surface before participating to oxidation and reduction reactions, result in hinder their practical applications [[10], [11], [12], [13]]. Up to now, numerous approaches have been followed to control or at least reduce these limitations. Some of these effective approaches are doping with other metals [14,15], adding co-catalyst [16,17], and fabricating heterojunction [18,19], to offer high PEC efficiency. The remarkable aspect of preparing a heterostructure system is that coupling the two semiconductor band gaps would generate appropriate band gap positions by aligning the Fermi levels at the interface. This approach has approved total improvement in the PEC activity of a system. Heterostructure systems of combining semiconductors lead to extended light absorption beyond the Ultraviolet (UV) spectrum, offering more photon absorption, tuning band alignment, and providing a large portion of exciting charge carriers for continuous reactions, which are migrating from small band gap semiconductors to large band gap semiconductors, resulting in sufficient separation and transport efficiencies [[20], [21], [22], [23]]. They form a protective layer from one to the other to avoid electric losses and increase the kinetic reactions leading to long-term stability. Due to that, they can reduce the chances of the charge carrier's recombination that occurs rapidly at surface states in a single photocatalyst.

Tungsten oxide (WO_3_) has been explored as a good photocatalyst that can emerge with other materials in various applications. The material exhibits excellent optical and electronic properties with a band gap between 2.7 and 2.9 eV, extending light absorption to 500 nm, inhibiting charge recombination, and promoting the performance of PEC water splitting. These features make tungsten oxide a promising compound in a composite, which cannot be found in single-component photoelectrodes [24,25]. Choosing an appropriate design strategy for forming a heterostructure composite plays an important role in the interfacial properties for sufficient charge transfer [26].

Castro et al.(2017) constructed WO_3_/TiO_2_ heterostructure as powder and films by adding WO_3_ with different rations in TiO_2_ structure and estimated band gap energy of the optimal sample of WO_3_/TiO_2_ - 40 wt% to be 3.23 eV [22]. He synthesized heterostructured nanoparticles using a hydrothermal route and then prepared a paste of produced nanoparticles to coat thin films. PEC measurements depicted an increase in photocurrent density for optimal films by 17 % with charge carriers' density of 1.59 × 10^20^ compared to those found in bare WO_3_. These were mainly ascribed to the aim of hole scavengers, optimized bandgap edge properties, and high light absorption response of produced films. Francesca et al. (2022) synthesized WO_3_/TiO_2_ phase space heterostructure films as single-phase and multi layers using an aerosol-assisted Chemical Vapor Deposition route [27]. He studied the impact of the thickness on the band gaps of produced films; it increased with increasing thickness. Also, he measured the photocurrent density for front and back irradiation and estimated it to be 0.87 mA cm^−2^ and 0.94 mA cm^−^ for WO_3_-30mL/TiO_2_ in 10 min and WO_3_-80mL/TiO_2_ in 3 min, respectively. That informed the system's synergetic improvements were attributed to the beneficial impact of a junction in separating photogenerated charge carriers. Sitaaraman et al. prepared WO_3_/BiVO_4_ heterojunction films using a spin coating technique with several layers of BiVO_4_ on WO_3_ surface [28]. In their study, the optimized system comprised 5 layers from each WO_3_ and BiVO_4_, presenting a large portion of the visible spectrum with more efficient charge migration than other counterpart photoanodes. As a result, overall water splitting reactions improved with a photocurrent density of about 0.64 mA cm^−2^ at 1.23V vs RHE. Sadaf group et al. fabricated an n-p heterojunction WO_3_/BiFeO_3_ with a measured thickness of ∼338 nm using a sol-gel spin coating technique [21]. They observed that excellent characterizations were recorded upon the used sol preparation of 2-methoxyethanol as a solvent and Diethanol amine as additives for obtaining the pure phase of BiFeO_3_ powder under temperature control. Photocurrent density for the system was measured at 2V vs Ag/AgCl at 35.2 mA cm^−2^ due to facilitated charge carries and prolonged carries lifetime.

The present study endeavors to elucidate the growth dynamics of the heterostructure WO_3_/TiVO_4_, aiming to fabricate a high-efficiency n-n photoanode through a sequential application of hydrothermal and spray pyrolysis techniques. A leading approach in band gap engineering is strategically integrating a broadband gap material (WO_3_) with a narrow band gap counterpart (TiVO_4_) to form a heterostructure [29]. This method notably augments light absorption in the visible spectrum and fosters enhanced charge separation [30]. In our experimentation, a triclinic phase of WO_3_ film was synthesized utilizing the hydrothermal method. Building upon our prior research [31], TiVO_4_ was deposited on the WO_3_ film, followed by specific thermal treatments after each procedure, culminating in forming the WO_3_/TiVO_4_ heterostructure. This work demonstrated using two straightforward hydrothermal and spray pyrolysis techniques for constructing an n-n type of integrated heterostructure photoanode. Based on the above features of WO_3_, it exhibits higher positive conduction and valence bands, making it easier to transport electrons from TiVO_4_ to WO_3_. Oppositely, the holes are transported from under layer WO_3_ to the upper layer of TiVO_4_. WO_3_ acts as an electron transfer layer to increase the efficiency of charge transport. It combines with TiVO_4_ to produce a type II heterostructure, while the upper layer of TiVO_4_ can be a light absorber. Comprehensive analysis of the resultant photoanode's electronic attributes, including band edge positions, photocurrent density, onset potential, and impedance, was conducted to ascertain the superior performance of the photoelectrochemical (PEC) water-splitting activity inherent to the system.

## 2 Materials and methods

Materials**.** In the fabrication of a WO₃/TiVO₄ heterostructure thin film, reagents, namely vanadium acetylacetonate, titanium isopropoxide, ethanol, trifluoroacetic acid, tungsten chloride, and nitric acid, were procured from Merck Life Science Products (U.K.) and employed without additional purification.

Fabrication of WO_3_ photoanode Tungsten oxide (WO₃) photoanodes was fabricated using a hydrothermal method. Specifically, a solution was formulated by dissolving tungsten chloride (0.02 M) in a 1:1 (v/v) blend of ethanol and deionized water. The pH of the solution was adjusted to 2 using nitric acid, followed by overnight stirring to achieve homogeneity. The resultant homogeneous solution was then transferred to a Teflon-lined autoclave. The conductive side of the pristine fluorine-doped tin oxide (FTO) glass substrates, each measuring 1 cm × 1 cm, was positioned downward, in close proximity to the stainless steel wall of the autoclave. The hydrothermal process was executed at 160 °C for 6 h, facilitating the growth of WO₃ films on the substrates. Post-synthesis, the coated substrates underwent annealing at temperatures of 500 °C, 550 °C, and 600 °C for 2 h in a muffle furnace before being allowed to cool to ambient conditions; produced films have pointed out WT_500 °C_, WT_550 °C_ and WT_600 °C_, respectively. The initial annealing temperatures were selected according to the previous reports [[30], [31], [32]].

## 3 Fabrication of WO_3_/TiVO_4_ photoanode

Titanium vanadate (Ti–V–O) photoanodes were fabricated via spray pyrolysis, as delineated in our prior work [33]. Briefly, a precursor solution was prepared through the dissolution of vanadium acetylacetonate and titanium isopropoxide in 15 mL of ethanol. This solution was supplemented with 0.05 mL of trifluoroacetic acid and subjected to a stirring period lasting 2 h. Subsequently, the prepared solution was aerosolized onto a pre-existing WO_3_ layer, possessing dimensions of 1 cm × 1 cm, while maintaining the substrate temperature at 250 °C. Post-deposition, the coated substrates underwent an annealing process at 600 °C for a duration of 2 h within a muffle furnace. Following thermal treatment, the samples were allowed to cool to ambient conditions in an open-air environment.

**Materials Characterisations.** To elucidate the structural composition and phases of the WO_3_/TiVO_4_ thin film, a Bruker D8 X-ray diffractometer (XRD) was employed, utilizing Cu-k_α_ (λ = 0.154 nm) radiation. The morphological characterization of the thin film was conducted using a TESCAN VEGA3 scanning electron microscope (SEM) equipped with energy-dispersive spectroscopy (EDS) provided by Oxford Instruments. Additionally, structural analysis included high-resolution transmission electron microscopy (HR-TEM), selected area electron diffraction (SAED), and scanning transmission electron microscopy (STEM) using the JEOL JEM-2100F TEM operating at 200 kV. X-ray photoelectron spectroscopy (XPS) measurements were executed with a Thermo NEXSA XPS instrument featuring a monochromated Al kα X-ray source (1486.7 eV). Thin film data acquisition occurred under a pressure below 10^−8^ Torr at a room temperature of 294 K. CasaXPS v2.3.20PR1.0 software facilitated data analysis, with calibration executed using the C1s peak at 284.8 eV. Furthermore, the diffuse reflection spectra of the thin films were acquired utilizing PerkinElmer's UV-VIS-NIR UV-3600 Plus spectrophotometer.

In the context of PEC analysis, WO_3_/TiVO_4_ photoanodes were employed in tandem with the Metrohm Autolab (PGSTAT302 N) workstation, featuring three-electrode compartments. The electrochemical test utilized a 1 M aqueous solution of NaOH with a pH of 13.6 as the electrolyte. Conversely, the reference electrode comprised a saturated aqueous solution of Ag/AgCl in KCl. Light intensity equivalent to 1 SUN condition (100 mW/cm^2^) was generated through a Newport setup, employing a 300 W xenon lamp equipped with an AM 1.5 filter and a 420 nm cut-off filter to eliminate ultraviolet radiation.

The voltage of the photoanode, referenced to Ag/AgCl, was meticulously monitored employing a scan rate of 0.01 V/s at a temperature of 25 °C. The recorded measurements encompassed a range from negative to positive potentials (−0.3 V to +0.75 V) under varying conditions, including illumination, darkness, and intermittent chopping. Subsequent to data acquisition, all potentials were transformed to a reversible hydrogen electrode (RHE) potential using the Nernst equation (Equation (1)).

(1) E_RHE_ = E_Ag/AgCl_+ 0.0591(pH) + 0.1976 V

Electrochemical Impedance Spectroscopy (EIS) analysis was conducted within a frequency range of 10^−2^ to 10^5^ Hz. The experiments were carried out utilizing a 1 M NaOH aqueous solution under illumination at 1 SUN (100 mW/cm^2^) and a fixed pH concentration of 13.6. The determination of the photoanode's flat band potential (Vf_b_) and dopant concentration (ND) was accomplished through the application of the Mott-Schottky equation, as outlined by the formula (Equation (2)),

(2) $${\left(\frac{1}{C}\right)}^{2}=\frac{2}{\epsilon_{r}\epsilon_{0}A^{2}\mathrm{e}N_{D}}\left(v-v_{fb}-\frac{K_{B}T}{e}\right)$$

Where C is the space-charge capacitance, *ε*₀ is the permittivity of vacuum, ε_r_ is the relative permittivity of a material, A is the area of the film, N_D_ is the carrier concentration, K_B_ is the Boltzmann constant, T is the operating temperature, e is the electronic charge, V is the applied potential, and V_fb_ is the flat band potential which is estimated through linear fit in Mott-Schottky plot.

## 4 Results and discussions

### 4.1 Characterization of photocatalysts thin film

**Fig. 1:**
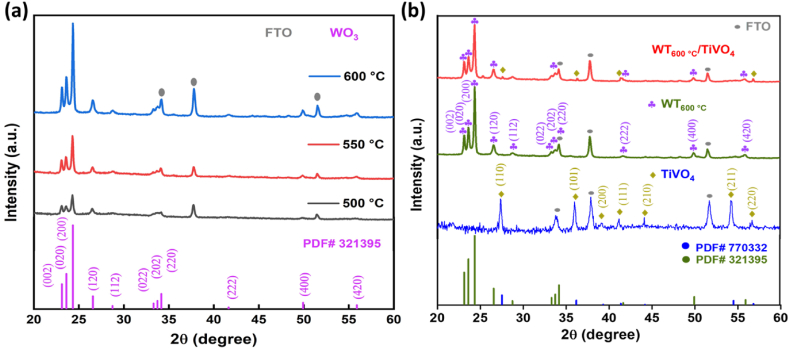
XRD pattern of (a) WO_3_ as a function of annealing temperatures, (b) bare TiVO_4_, WT_600 °C_ and WT_600 °C_/TiVO_4_ photoanodes deposited on FTO glass.

XRD Analysis: Fig. 1(a) represents X-ray diffraction peak patterns of WO_3_ as a function of annealing temperatures. All samples have distinct peaks assigned to triclinic structure with corresponding predominant peaks at crystal planes of (002), (020) and (200), JCPDS no. 32–1395 [34]. It is clear that the samples ‘s crystallinity became high with the increase of annealing temperatures. One can see that the sample annealed at 600 °C has shown the highest crystallinity. Above 600 °C, films exhibited significant cracks throughout the film due to exposure to the two heat treatments after each procedure [35]. To determine the change of XRD patterns of the heterostructure composite compared with two other bare films, X-ray diffraction peak patterns of TiVO_4_, WT_600 °C_ and WT_600 °C_/TiVO_4_ thin films were investigated, as shown in Fig. 1(b). The bare TiVO_4_ thin film displays three dominant peaks, which are sequentially corresponded with (110), (101) and (220) crystal planes of tetragonal structure (JCPDS no. 01–770332) [33]. While, WT_600 °C_/TiVO_4_ heterostructure thin film demonstrates distinct peaks of both tetragonal structure and triclinic structure with no impurity phases, which indicates that no significant change in the diffraction peaks of the triclinic WO_3_ structure after sprayed the top layer of TiVO_4_, and then annealing at 600 °C for 2 h. On the other hand, the absence of some peaks of TiVO_4_ within the composite diffraction pattern or lower intensities may be because the sprayed TiVO_4_ is thinner than the WO_3_ layer [36].

**Fig. 2:**
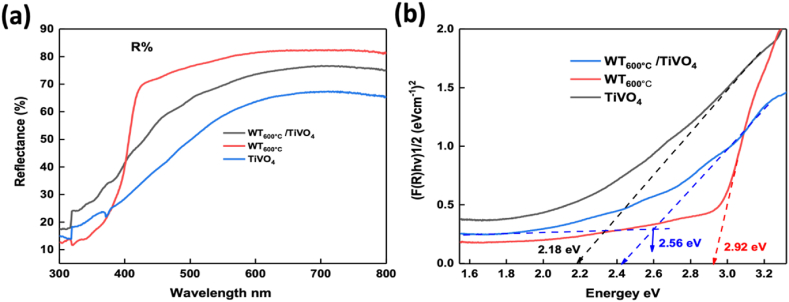
(a) Reflectance spectra, (b) and the corresponding band gaps of WT_600 °C_, TiVO_4_ and WT_600 °C_/TiVO_4_ photoanodes.

**Optical Analysis:** To investigate the band gaps and reflectance characteristics of the prepared photoanodes, we conducted diffuse reflectance measurements and corresponding band gap assessments, as illustrated in Fig. 2. The reflectance spectra, depicted in Fig. 2(a), indicated maximal values for the WT_600°C_ photoanodes and minimal values for the TiVO_4_ counterparts. Notably, the WO_3_/TiVO_4_ heterostructure exhibited intermediate reflectance between the two bare films, with a reduction compared to the observed reflectance in the WO_3_ film [37]. The determination of corresponding band gap values was undertaken through the utilization of the Kubelka-Munk equation [38]. The estimated band gap values for WT_600 °C_, TiVO_4_, and WT_600 °C_/TiVO_4_ are established at 2.92 eV, 2.18 eV, and 2.56 eV, respectively (Fig. 2(b)). It is evident that these estimations demonstrate a favourable concordance with previously reported band gap values for WO__3__ and TiVO__4__ photoanodes [39].

**Fig. 3:**
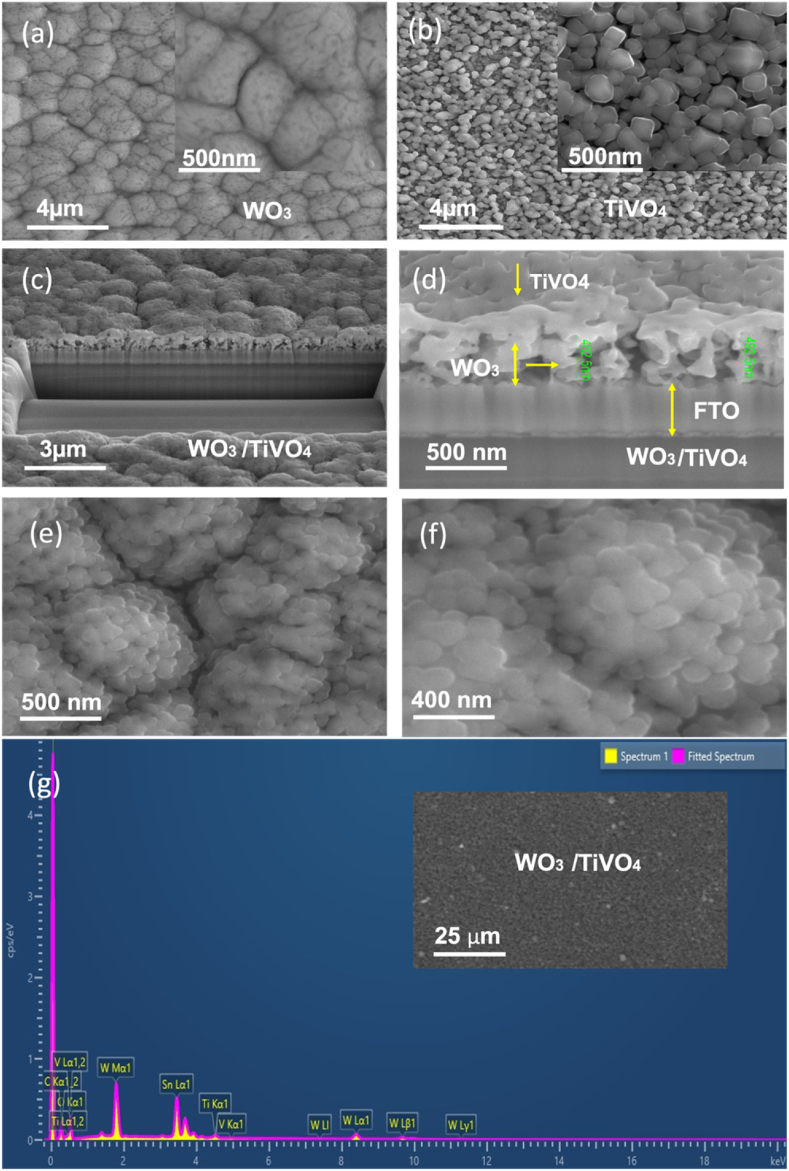
SEM microstructural images of (a) bare WT_600 °C_, (b) bare TiVO_4_, (c–d) heterostructure WT_600 °C_/TiVO_4_ cross-section images with different magnifications, (e–f) surface top view SEM images, and (g) EDS spectrum of the WT_600 °C_/TiVO_4_ photoanode.

Microstructural Analysis: Fig. 3(a–f) present scanning electron microscopy images depicting diverse photoanodes at varying magnifications. Fig. 3(a) illustrates top-view images at 4 μm and 500 nm scales, revealing a porous planar surface of a WO_3_ film annealed at 600 °C for 2 h. This film comprises moderate and large interconnected grains, with complete coverage across the entire surface of the FTO substrate. In Fig. 3(b) the bare tetragonal TiVO_4_ particles grew uniformly on FTO glass with smooth distribution and were highly porous structures in nature, which is a good correlation with previous work [33]. Cross-section SEM images of microstructure as prepared WO_3_/TiVO_4_ at different magnifications are shown in Fig. 3(c) and (d). The findings suggest a uniform film formation characterized by the agglomeration of sprayed TiVO_4_ particles, leading to the development of structures reminiscent of flower-like adherences. This phenomenon contributes to heightened porosity within the agglomerations. Notably, both layers exhibit clear conformity, without discernible separation. The presence of the FTO layer is conspicuously evident, affirming the establishment of a heterostructure thin film with a total thickness measuring 492 nm.

Furthermore, the TiVO₄ particles, deposited as the top layer, exhibited discernibly reduced dimensions and augmented interconnectivity in comparison to their counterparts within the untreated film. This morphological transformation confers a notable advantage by facilitating a more efficient migration of charge carriers toward the junction, thereby amplifying surface reactions, as depicted in Fig. 3 (e,f). Therefore, the marked enhancement in PEC activity of the heterostructure can be primarily attributed to its porous nature, which forms channels amidst the agglomerated particle groups on the upper layer, expanding its surface exposure to the active region of the underlying thin film [28]. Fig. 3(g) shows the EDS spectrum with distinct peaks of WT_600 °C_/TiVO_4_ heterostructure deposited on the FTO substrate, indicating the presence of the composite's elements of Ti, V, W, Sn, C and O.

**Fig. 4:**
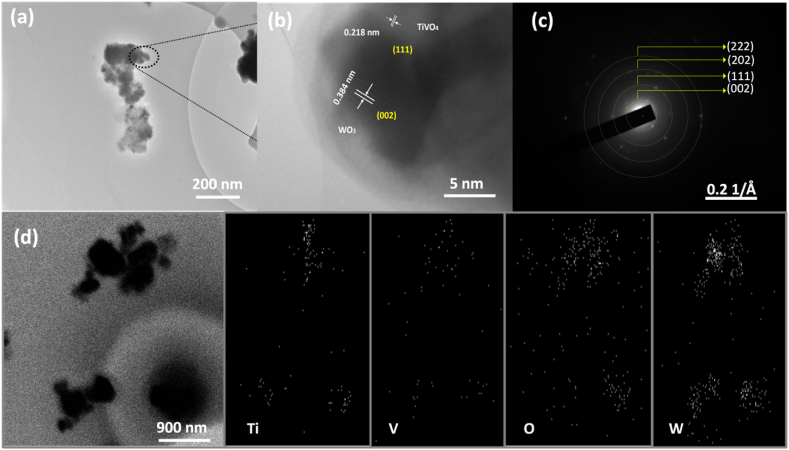
TEM (a) bright field image, (b) corresponding HRTEM, (d) SAED and (e) STEM images of the WT600 °C/TiVO4 thin film.

The TEM and high-resolution TEM micrographs in Fig. 4(a and b) revealed the lattice fringes corresponded to an interplanar distance of 0.218 nm and 0.384 nm for the (111) plane of TiVO_4_ and (002) plane of WO_3_, respectively, indicating a tight contact is formed between them leading to a high crystallinity. To ensure the lattice planes and the construction growth of heterostructure composite, SAED was carried out and shown in Fig. 4**(c)**. Fig. 4(d) shows STEM images of WT_600 °C_/TiVO_4_ that confirm the existence of Ti, V, O and W elements, which are consistent with SEM images.

**Fig. 5:**
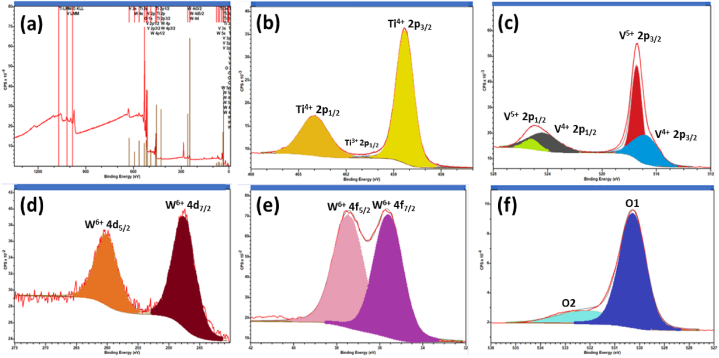
(a) XPS survey spectrum of the heterostructure WT_600 °C_/TiVO_4_ the thin films on FTO glass, (b) XPS spectrum of spin-orbit deconvoluted peaks of Ti 2p, (c) V 2p, and (d) W 4d, (f) W 4f and (f) O1s levels, respectively.

X-ray photoelectron study: To gain a comprehensive understanding of the WT_600 °C_/TiVO_4_ composite formation, an analysis of surface composition was conducted through X-ray Photoelectron Spectroscopy (XPS) integrated peak area analysis, as illustrated in Fig. 5. Calibration of all binding energies was executed using the contaminant carbon (C 1s = 283.4eV) as the reference standard. In Fig. 5(a), the surface spectrum of the sample is presented, confirming the detection of core-binding energy states for Ti 2p, V 2p, O 1s, W 4d, and W 4f, elucidating the elemental composition of the composite. Characteristics of the Ti 2p spectrum are elucidated in Fig. 5(b), wherein the two predominant spin-orbit split peaks of Ti 2p_1/2_ and Ti 2p_3/2_ were identified at 463.7 and 457.8, respectively. This observation results in a spin-orbit splitting energy of 5.8 eV, indicative of the characteristic +4-oxidation state of Ti within the TiVO₄ structure. Notably, no discernible peak corresponding to Ti^3+^ was identified, likely attributed to the partial reduction of Ti^4+^ resulting in oxygen vacancies. This suggests that the TiVO₄ component in the composite exclusively contributed to Ti^4+^ [40]. In Fig. 5(c), the robust hybridization between V 2p and O 1s states is depicted, elucidating the diverse oxidation states of V 2p during the formation of TiVO₄. Notably, the prevalence of the +5 oxidation states, among V's three principal oxidation states (+5, +4, and +2), is apparent. The V 2p_3/2_ satellite peak, situated approximately at 527 eV, is positioned between the V 2p_1/2_ and O 1s spectral features. Simultaneously, the V 2p_1/2_ satellite peak is situated on the higher binding energy side of the O 1s peak, coexisting with states associated with the concentration of –OH [41]. Contrastingly, for the +4 oxidation states, the V 2p_3/2_ and V 2p_1/2_ were identified at 515.8 eV and 523.01 eV, respectively. The most stable V^5+^ oxidation states were discerned at 516.6 eV and 524.1 eV, corresponding to the spin-orbit binding energies of V^5+^ 2p_3/2_ and V^5+^ 2p_1/2_ states [41].

The interpretation of the XPS data revealed minimal V^2+^ peaks, which were nearly undetectable during peak fitting. Nevertheless, the concurrent presence of V^4+^ and V^5+^ peaks suggest the existence of vanadium ions within the TiVO_4_ sample. The core-level XPS spectrum of W 4d in Fig. 5(d) provides clear evidence of the presence of WO₃ in the composite, confirming the +6 oxidation states of tungsten. This observation is further substantiated in Fig. 5(e), where the binding energy positions of 4f_7/2_ and 4f_5/2_ are prominently displayed. The identification of a 4:3 peak ratio of W (4f_7/2_) to W (4f_5/2_) with a spin-orbit splitting doublet value of 2.12 eV underscores the oxidation state of W as +6. Additionally, a broad peak of W (5p_3/2_) at approximately 41.8 eV solidifies the exclusive presence of W in its +6 oxidation state. The asymmetry observed in the W 4f_7/2_ peak suggests the potential for oxygen deficiency, leading to the formation of WO_3-x_. In contrast, the deconvolution of the asymmetric O 1s XPS spectrum in Fig. 5(f) into two components revealed distinct characteristics. The dominant O1 component aligns with O^2−^ ions, while the O2 component, positioned at a higher binding energy, may be attributed to chemisorbed oxygen or the hydroxyl group (-OH). The latter could potentially result from moisture adsorption during synthesis. However, this oxygen deficiency has a notable impact on electronic transport properties due to the introduction of donor electronic states [42,43].

In response to identified limitations within single-component photocatalytic systems, extensive research efforts have been directed toward optimizing the configuration of photocatalysts. One prominent strategy involves the development of type-II heterojunction nanocomposites utilizing two distinct semiconductors. This deliberate design aims to augment charge separation efficiency and elevate overall photocatalytic performance. The intentional establishment of heterojunction-type photocatalytic systems induces spatial isolation of electrons and holes, effectively extending the lifetime of photogenerated carriers. Subsequent optical and structural characterizations have confirmed the successful formation of the WO_3_–TiVO_4_ composite. This composite signifies the creation of a heterojunction, thereby indicating the potential for generating coupled colloidal structures. Under illumination, such structures prompt a response in the second semiconductor at the interface, rendering them active under visible light irradiations.

The amalgamation of semiconductor photocatalysts results in exceptional photocatalytic activity, primarily attributed to the mitigation of charge recombination and the expansion of the energy range of photo-excitation. These advancements hold significant promise for advancing the field of photocatalysis, particularly in the context of visible light-driven reactions.

**Fig. 6:**
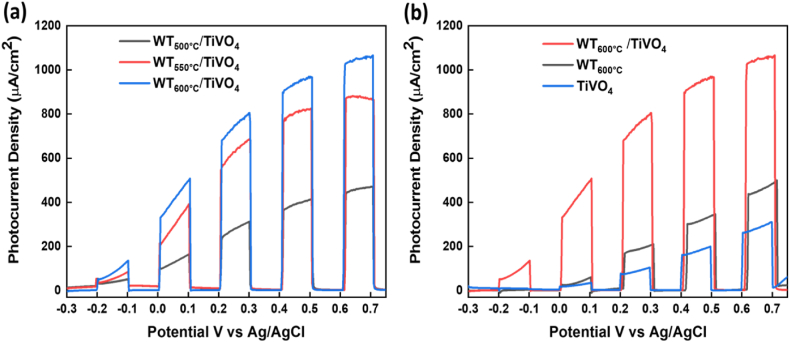
(a) Linear Sweep Voltammetry (LSV) plots of current density versus potential (referenced to Ag/AgCl) under intermittent illumination at an intensity of 100 mW/cm^2^ in a 1 M NaOH electrolyte (pH 13.6). The plots represent a heterostructure film of the WO₃ layer annealed at temperatures of WT_500 °C_, WT_550 °C_, and WT_600 °C_ for 2 h, integrated with TiVO₄. (b) LSV plots under similar conditions for bare TiVO₄, bare WT_600 °C,_ and the heterostructure WT_600 °C_/TiVO₄ films.

**Photoelectrochemical (PEC) measurements of WO**_**3**_**/TiVO**_**4**:_**Heterostructure thin films:** The benefit of the heterojunction in charge transfer has been evident by the photoelectrochemical measurements. To evaluate the PEC activities of various WO_3_/TiVO_4_ composites**,** where WO_3_ layers were a function of annealing temperatures, the linear sweep voltage (LSV) was implemented at a scan rate of 0.01V/s and recorded under chopped condition over the potential range - 0.3 to 0.75 V, as shown in Fig. 6(a). LSV plots revealed photocurrent (μA) vs potential, V (Ag/AgCl) of WO_3_ annealed at different temperatures as under layer, thus coupled with TiVO_4_ layer. The highest photocurrent density was observed upon WT_600 °C_/2hrs)/TiVO_4_ film, reaching 0.740 mA at 1.23 V vs. RHE and a maximum value of 1.10 mA at 1.7 V vs RHE. With simulated solar irradiation, the photocurrent densities of two films, WT_550 °C_/2hrs)/TiVO_4_ and WT_500°C_/2 h)/TiVO_4_ were recorded to be 0.650 mA and 0.300 mA at 1.23 V vs. RHE, respectively. As can be seen, this result concludes the annealing temperature impact on triclinic WO_3,_ where 600 °C exhibited the highest and maximum photocurrent density among other annealing temperatures.

Fig. 6(b) displays LSV plots in comparison of three different films, bare TiVO_4_, bare WT_600 °C_ and WT_600 °C_/TiVO_4,_ to investigate the advantages of the integration of WO_3_ as under layer with bare TiVO_4_. Here, we employed WO_3_ annealed at 600 °C as plotted previously and exhibited the highest photocurrent density coupling with tetragonal particles of TiVO_4_. TiVO_4_ and WO_3_ photocurrent densities in both bare films were observed at 1.23 V νs. RHE to be 0.080 mA and 0.200 mA, respectively. These results are close and agreed with previous reports [42]. Coupling WO_3_ with TiVO_4_ raised photocurrent density about nine times that recorded in bare TiVO_4_. This excellent improvement is attributed to a good designation of WO_3_ with TiVO_4_ to construct a heterostructure for PEC application [[44], [45], [46]]. WO_3_ as a promising candidate in heterostructure strategy showed an ideal role of broadening the light absorption of TiVO_4_ by creating suitable band gaps, appropriate band positions and enhanced charge carriers transfer that allowed the bare film to straddle uphill water reactions.

**Fig. 7:**
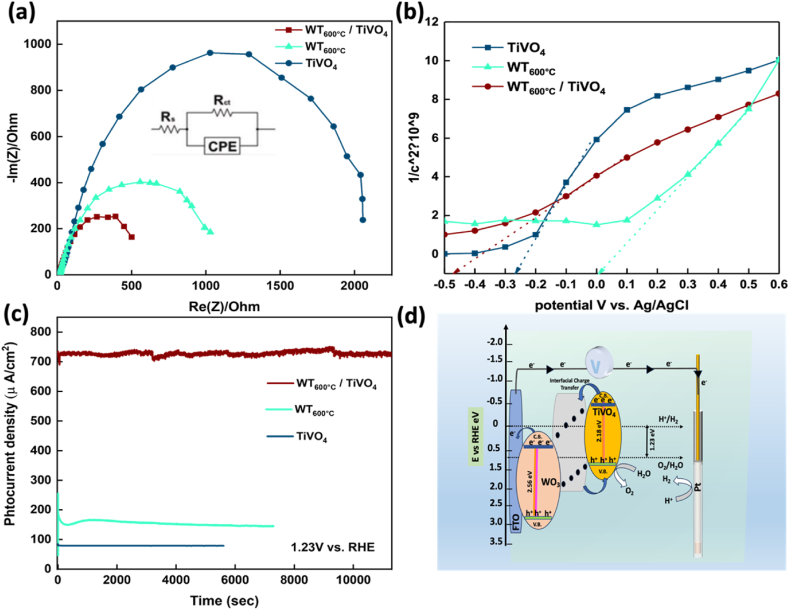
(a) Nyquist plots, (b) Mott Schottky and (d) the stability plots of prepared TiVO_4_ and WT_600 °C_/TiVO_4_ films, (c) schematic illustration of possible charge transfer of WT_600 °C_/TiVO_4_ heterostructure film.

**Electrochemical Impedance Spectroscopy (EIS) Analysis:** Electrochemical impedance spectroscopy is a powerful measurement for investigating the charge kinetics at an interface of any system in terms of the appropriate equivalent circuit for the system. EIS provides an overall analysis of the charge carriers' transfer mechanism and their resistance at the interface between the working electrode/electrolyte over the chosen circuit. Fig. 7(a) displays Nyquist plots of bare TiVO_4_, WO_3_ and WT_600_^o^_C_/TiVO_4_ films under illumination in the 10^−2^ Hz–10^5^ Hz frequency range with the equivalent circuit, shown in Fig. 7(a) (inset). Estimated resistances in the equivalent circuit indicate R_s_ where displays film resistance of the charge collector of the substrate (FTO), CPE represents the constant phase element, and R_ct_ indicates to the charge carrier transfer resistance at the interface (electrode/electrolyte). The heterostructure film demonstrated the smallest semi-circle at high frequency, corresponding to a decreased interfacial resistance compared to the bare TiVO₄ and WT_600 °C_ photoanode. Specifically, resistance values of 668.6 Ω, 1149 Ω and 2027 Ω were measured for the heterostructure, the bare TiVO₄ and WT_600 °C_ photoanodes, respectively [43]. This notably reduced charge transfer resistance and surface state recombination, as inferred from the Electrochemical Impedance Spectroscopy (EIS) plot for the heterostructure system, can be attributed to the high electrical conductivity of WO₃, as previously reported [47,48]. The generated built-in potential caused by band bending of the heterogenous structure creates a large drift of charges, resulting in a homogeneous charge distribution on the working electrode's surface and across the interface. Consequently, the electrons flow easily to the collector layer of WO_3_, reducing the trap surface states, and then transferring through an external circuit. While the holes move to TiVO_4_ to enable the oxidation reaction. This phenomenon culminates in forming an intrinsic electric field, subsequently reducing electrochemical overpotential for maximising photocurrent density and shifting onset potential negatively.

Fig. 7(b) displays Mott Schottky plot of WT_600 °C_/TiVO_4_ heterostructure compared to bare TiVO_4_ and WT_600°C_. Flat band potential for heterostructure was observed to be more negative than were recorded in two bare films, which indicated band gap position shift with an estimated value of −0.46 V νs. Ag/AgCl V νs. The valence and conduction bands of the composite were extracted from obtained (E_fb_) to be −0.54 V νs. RHE and +2.02 V νs. RHE. Others two bare films' flat band potentials were in a good correlation with previous reports [49]. As more, the concentration dopant (N_d_) was calculated from Mott Schottky equation (2), as estimated to be 9.2 × 10^20^ cm^−3^, higher than were observed in TiVO_4_ (7.7 × 10^20^ cm^−3^) and WT_600 °C_ (8.1 × 10^20^ cm^−3^) films [50]. In the heterostructure, the equilibrium state, determined by the distribution and concentration changes of photogenerated charge carriers across the coupled photoanodes, shifts the energy band-edge positions. These new positions are more favourable about the potentials of the two requisite evolution reactions. Consequently, this adjusted band gap facilitates the migration of electrons from the conduction band of TiVO_4_ to the WO_3_ layer.

Given that the conduction band of pristine TiVO_4_ is higher than that of pristine WO_3_, electrons can traverse the electrical circuit to the counter electrode (Pt) and participate in the water reduction reaction. Simultaneously, holes are transferred from the valence band of WO_3_ to TiVO_4_, where they react with water species [22,23]. Fig. 7(c) represents the stability of the photocurrent density of TiVO_4,_ WT_600 °C_ and the constructed nanostructure composite WT_600 °C_/TiVO_4_ under illumination condition, and is recorded at an applied bias potential of 1.23 V vs RHE. For the bare TiVO_4_ film, stable photocurrent reached 95 % of the original steady state photocurrent after 6000s, while the bare WO_3_ maintains only 85 % of photocurrent, indicating that the TiVO_4_ layer is employed as a protective layer in the PEC stability. Compared to the two bare films, the composite film elucidated greater stability for a long period (3 h), as shown by chronoamperometric measurements. As XPS showed oxygen vacancies during the composite creation, some noisy signs gradually were appeared in the photocurrent density of the composite throughout the test. These signs were probably caused by oxygen bubbles formed on the electrode [21]. Insufficient stability of a single photoelectrode occurs due to photocorrosion of an incohesive electrode inside a harsh electrolyte. By focusing on the electrode part, integrating stable trended layer/catalysts may promote stability by physically protecting the separated electrons under illumination. On the other hand, controlling the reactions inside different concentrated pH electrolytes by adding ions into a solution leads to long-term stability via boosting dissolution during the PEC operation [51].

Gas evolution, a critical aspect of photocatalysis, manifests in two distinct phases within separated photocatalysts. Specifically, hydrogen evolution reaction (HER) occurs on H_2_ evolution photocatalysts (HEP), while oxygen evolution reaction (OER) takes place on oxygen evolution photocatalysts (OEP). Despite the demonstrated competency of heterojunction-type semiconductor nanocomposites in facilitating efficient charge separation, a noteworthy compromise exists in the photocatalytic system's redox ability. This compromise arises from the migration of electrons and holes toward more electropositive CB and more electronegative VB potentials, attributed to the inherent nature of charge transfer, as illustrated in Fig. 7(d). To delineate the band edge levels of the films and establish a correlation between heterojunction formation and the observed enhancement in photocurrent, as evidenced by photocurrent-voltage measurements, the conduction band edge (E_CB_) can be estimated. This estimation involves a slight shift of the flat band potential (E_fb_) obtained from the Mott-Schottky plot. The determination of the valence band edge (E_VB_) is subsequently achieved concerning E_CB_, denoted as in Equation (3),

(3) E_VB_ = E_CB_ + E_g_

where Eg is extracted through diffuse reflectance spectroscopy (DRS) analysis. The energy diagram, derived experimentally, is depicted in Fig. 7(d) [22]. Based on the outcomes of this study, the formation of a Type II heterostructure between TiVO_4_ and WO_3_ is evident. This observation aligns closely with recent literature, highlighting the pronounced improvement in spatial charge separation and a concurrent enhancement in photocatalytic response. The results presented herein furnish persuasive arguments to explicate the underlying mechanisms operative at the electrode surface during photoassisted water splitting. Under simulated solar irradiation, WO_3_ generates electron-hole pairs owing to its distinctive electronic structure. The holes, positioned at the E_VB_ of WO_3_, undergo spontaneous injection into the E_VB_ of TiVO_4_. This process is facilitated by the work function and chemical potential difference between these semiconductors at the interface, indicative of a Type II band alignment. The established heterostructure demonstrates promise for advancing our understanding of fundamental processes governing photoassisted water splitting reactions.

Therefore, the main reason for improvements in PEC performance is the formation of a good structure of n-n heterostructure between selected oxide materials that shift the band gap position of the junction to an appropriate position, resulting in broadening light harvesting and facilitating charge carriers transfer [52]. Produced heterostructure comprises a staggered band structure, the most effective band for photocatalytic application. Here, the conduction band position of WO_3_ is lower than for TiVO_4_, allowing electrons to transport from the top layer to the down layer and vice versa for the holes. Charge carriers' transfer between layers creates a built-in electric field caused by potential difference, forming a depletion layer until reaching the equilibrium state with less barrier, which can facilitate charge transport through the interface. Photogenerated carriers transfer easily to the surface to participate in redox reactions [53]. Thus, the formation of n–n heterojunctions between WT_600 °C_ and TiVO_4_ components facilitated the transfer of charge carriers, which was proved by the photocurrent density and EIS analyses and observed in the impressive enhancement of the photocatalytic activity. Distinguishing the direct *Z-scheme* from the conventional type-II heterojunction can be achieved through radical trapping experiments, as the photogenerated carriers are strategically situated at distinct potential levels within these two systems. In light of recent investigations into the heterostructure formation of WO_3_-based thin-film photocatalysts, we have undertaken a comprehensive synthesis of the current comparative analysis encompassing select high-performing WO_3_– TiVO_4_ heterostructure composites. The outcomes, specifically addressing improvements in photocurrent density, have been summarized in Table 1 with relevance to their PEC application [28,43,50,55,56].

**Table 1:** Comparative study of some of the best performing WO_3_ - heterostructure composites and their photocurrent density improvements in PEC application.

Sl No.	Heterostructure Photocatalyst Thin Film	Photocurrent density at 1.23 V vs. RHE	Improvement in the Photocurrent density to counterpart film	Mechanism type	Reference
1	WO_3_/BiVO_4_	0.64 mA/cm^2^	1.6 times	n-n	[28]
2	WO_3_/NiCo_2_O_4_	0.84 mA/cm^2^	4 times	n-p	[42]
3	WO_3_/Ti–Fe_2_O_3_	2.15 mA/cm^2^	3.9 times	n-n	[49]
4	WO_3_/CuWO_4_	0.80 mA/cm^2^	4 times	n-n	[54]
5	WO_3_/Fe_2_O_3_	0.588 mA/cm^2^	5 times	n-n	[55]
6	WO_3_/TiVO_4_	0.740 mA/cm^2^	9 times	n-n	This work

### 4.2 Techno-economy perspective

Sunlight represents a compelling avenue for mitigating carbon dioxide emissions and ensuring a sustainable energy future. Projections indicate that over 50 % of global power generation will be derived from renewable sources by 2035, with solar energy constituting a significant portion [56]. This underscores untapped potential beyond current utilization, prompting research into economically viable technologies for solar energy conversion, storage, and utilization. The endeavor to photoelectrochemically or electrochemically diminish CO_2_ directly holds promise for generating products of greater value than hydrogen. However, numerous unresolved challenges impede progress, such as catalytic efficiency, selectivity, CO_2_ mass transport rates, and feedstock cost. Significant breakthroughs are imperative to attain economically viable costs for solar hydrogen production. Yet, the obstacles to achieving cost-competitiveness with prevailing large-scale thermochemical processes for CO_2_ reduction present even more formidable challenges. While particulate photocatalytic water splitting offers advantages, its industrial application faces limitations due to relatively low solar-to-hydrogen efficiency (STH), posing cost challenges. Presently, renewable hydrogen costs approximately US$5 per kg. Despite achieving 4 % and 20 % STH efficiencies for PEC and PV-EC hybrid systems at the laboratory scale, the economic appeal of photocatalytic water splitting using particulate photocatalysts remains significant [57]. Techno-economic analyses suggest that hydrogen production costs via PEC systems range from US$ 4.0 to US$ 10.4 per kg, depending on STH values and system lifetimes. In contrast, estimates for hydrogen production from solar water splitting using particulate photocatalysts range from US$ 1.6 to US$ 3.2 per kg. This underscores the cost advantage of particulate photocatalysts, which may decrease further with improved STH efficiency and device stability [58,59].

The challenge lies in bridging the gap between scientific advancements and practical technology for scalable and direct hydrogen production through solar-driven water splitting. Addressing low STH efficiencies, H_2_/O_2_ gas separation, and reverse reaction inhibition is crucial for large-scale implementation. While water-splitting panels show promise, obstacles like light-harvesting mechanisms, efficient charge separation, dual-cocatalyst strategies, and scalability issues must be overcome for future industrial applications. Researchers are urged to persistently dedicate efforts to propel solar water splitting from fundamental research to practical industrial use.

## 5 Conclusion

The fabrication of an n-n heterostructure consisting of WT_600 °C_/TiVO₄ photoanodes led to an augmentation in PEC activity. This improvement was attributed to the optimal band alignment, highlighting the significance of tungsten oxide as a prospective layer. The annealing temperatures applied to the tungsten oxide layer were found to influence the heterostructure's composition critically. The optimal annealing temperature for the WO₃ underlayer, which yielded the highest PEC performance, was identified at 600 °C. This temperature facilitated enhanced charge transport across the top layer and at the interface between the composite and electrolyte. The maximum photocurrent density for the WO₃ annealed at 600° WT_600 °C_/TiVO₄ composite was measured at 1.23 V vs RHE, registering at 0.740 mA/cm^2^. This value represents an approximately nine-fold increase compared to the TiVO₄ photoanode. The integration of WO₃ with the pristine TiVO₄ photoanode resulted in added enhancements by diminishing recombination rates and bolstering charge carrier transfer. This improvement showcases the merit of integrating two scalable and cost-effective methods. Transmission electron microscopy (TEM) images confirmed the presence of composite constituents with matching crystalline planes, free from lattice distortions, as corroborated by XRD patterns. Chronoamperometric analyses demonstrated the composite's commendable stability; the photocurrent density showed negligible degradation over a testing period of 3 h. This study establishes a foundation for a more comprehensive comprehension of the interfacial band structure within WO_3_ and TiVO_4_ heterojunction electrodes. The insights gained from this work may enhance the conceptualization and design of more effective photocatalytic systems.

## Data availability

Data associated with this study have not been deposited into a publicly available repository. Data will be made available on request to the corresponding author.

## CRediT authorship contribution statement

**Manal Alruwaili:** Writing – original draft, Methodology, Investigation, Formal analysis, Data curation, Conceptualization. **Anurag Roy:** Writing – review & editing, Validation, Methodology, Formal analysis. **Mansour Alhabradi:** Investigation, Formal analysis, Data curation. **Xiuru Yang:** Investigation, Formal analysis, Data curation. **Hong Chang:** Investigation, Data curation. **Asif Ali Tahir:** Writing – review & editing, Validation, Supervision, Resources, Project administration, Funding acquisition.

## Declaration of competing interest

The authors declare that they have no known competing financial interests or personal relationships that could have appeared to influence the work reported in this paper.
